# Prognostic Significance of Cyclins A2, B1, D1, and E1 and *CCND1* Numerical Aberrations in Oral Squamous Cell Carcinomas

**DOI:** 10.1155/2018/7253510

**Published:** 2018-03-27

**Authors:** Luís Silva Monteiro, Márcio Diniz-Freitas, Saman Warnakulasuriya, Tomás Garcia-Caballero, Jerónimo Forteza-Vila, Máximo Fraga

**Affiliations:** ^1^Institute of Research and Advanced Training in Health Sciences and Technologies (IINFACTS), University Institute of Health Sciences (IUCS), CESPU, CP 4585-116 Paredes, Portugal; ^2^Medical-Surgical Dentistry Research Group (OMEQUI), Health Research Institute of Santiago de Compostela (IDIS), University of Santiago de Compostela, CP 15782 Santiago, Spain; ^3^Oral Medicine Department, The Dental Institute, King's College and the WHO Collaborating Centre for Oral Cancer, London SE5 9RW, UK; ^4^Morphological Sciences Department, School of Medicine, University Clinical Hospital, University of Santiago de Compostela, CP 15782 Santiago, Spain; ^5^Instituto Valenciano de Patología, Universidad Católica de Valencia y Área Mixta de Investigación Oncológica (Centro de Investigación Príncipe de Valencia-UCV), CP 46012 Valencia, Spain; ^6^Pathology Department, School of Medicine-University Clinical Hospital, University of Santiago de Compostela, CP 15706 Santiago, Spain

## Abstract

We analysed the expression of cyclins A2, B1, D1, and E1 by immunohistochemistry and numerical aberrations in *CCND1* gene by fluorescence in situ hybridization technique in 67 primary oral squamous cell carcinomas (OSCC). Cyclin A2 expression was observed in 54 (83.1%) tumours, cyclin D1 in 58 (89.2%), cyclin B1 in 39 (60%), and cyclin E in 21 (32.8%). *CCND1* region analysis revealed 26 (43.3%) tumours with the presence of numerical aberrations which were correlated with cyclin D1 high expression (Rho = 0.48; *p* < 0.001). Twenty-nine (45.3%) tumours were classified as high proliferative tumours assessed by Ki-67 protein expression and correlated with tumours with high expression of cyclin A2 (Rho = 0.30; *p* = 0.016) and cyclin B1 (Rho = 0.37; *p* = 0.003). In multivariate analysis for an overall five-year survival (OS), we found an adverse independent prognostic value for cyclin A2 high expression (*p* = 0.031) and for advanced tumour stage (*p* < 0.001). Our results confirm that several cyclins are commonly expressed in OSCC. *CCND1* gene is abnormal in more than one-third of the cases and is frequently associated with cyclin D1 high expression. Moreover, cyclin A2 high expression is an independent indicator of worse OS suggesting that this protein may serve as a reliable biological marker to identify high-risk subgroups with poor prognosis.

## 1. Introduction

Oral cancer remains a significant cause of morbidity and mortality, with 529,451 new cases and 292,289 estimated deaths annually worldwide in 2012 [1]. Over 90 percent of cancers of the oral cavity are represented by oral squamous cell carcinomas (OSCC). Despite technological advances in the detection and management of oral cancer during the last few decades, many centres still report low survival rates (~50%) [2].

It is believed that oral carcinogenesis involves a series of genetic alterations that frequently involve normal cell cycle control proteins such as cyclins and their enzymatically active partners, the cyclin-dependent kinases (CDK) [3, 4]. The discovery of major proteins involved in these alterations could identify new molecular markers that may serve as prognostic markers of OSCC and help in developing more precise treatment plans for these cancers [3, 5].

Cyclins are divided into two groups based on their function: the G1 cyclins (C, D, and E), regulating the passage of cells through the G1 phase and their entry into the S phase, and the mitotic cyclins (A, B) [3, 6]. The *CCND1* is a proto-oncogene located in chromosome 11q13 that encodes cyclin D1. This protein binds and activates CDK4 and CDK6, leading to phosphorylation of pRb driving the cell cycle from the G1 to the S phase. *CCND1* amplification and overexpression have been reported to be frequent events in several tumours including head and neck cancers [7, 8]. Several studies have reported the correlation between cyclin D1 overexpression and *CCND1* amplification, lymph node metastasis, local recurrence, advanced histological grade (G2/G3), and poor survival [9, 10]. Cyclin A is required for DNA synthesis during the S phase and progression through the G2/M transition. Cyclin A overexpression has been found to be an adverse prognostic factor in oral premalignant and malignant lesions [6, 11, 12]. Cyclin E is expressed in the middle of the G1 phase and ends at the beginning of the S phase. High levels of cyclin E1 may lead to accelerated G1/S transition and in an elongated S phase duration, resulting in an increased chromosomal instability [13]. Overexpression of cyclin E1 has been reported in several cancers including nasopharyngeal carcinomas with an influence on prognosis [14]. Cyclin B1 is crucial to drive epithelial cells into the mitosis phase. Uncontrolled cyclin B1 expression may result in premature entry into mitosis, abnormal cell proliferation, and neoplastic transformation. Cytoplasmic overexpression of cyclin B1 has been reported in several cancers including head and neck cancers and is related with advanced histological grade and local recurrence of these cancers [15].

The aim of the current study was to evaluate the expression of cyclins A2, B1, D1, and E1 and CCND1 gene status in a single cohort of patients with oral squamous cell carcinomas (OSCC) and relate them to clinical-pathologic characteristics and patient outcome.

## 2. Patients and Methods

### 2.1. Patient Population

In this observational study, we included 67 patients newly diagnosed and treated for primary OSCC (ICD: C01–06) at the Clinical University Hospital of Santiago de Compostela (CUHSC) (Spain), between 1995 and 2003. The study was undertaken following the approval of the institutional ethical board of the hospital and performed in accordance with the Declaration of Helsinki. We followed the methods of Monteiro et al. [16]. Briefly, clinical information was obtained from the patient's records, including gender, age of the patient, tumour site, stage classification (7th edition of American Joint Committee on Cancer) [17], treatment performed (Table 1), surgical margin status, and follow-up information. On a new 4 *μ*m hematoxylin-eosin (HE) section, we revaluated histological diagnosis, tumour differentiation grade (WHO 2005) [18], and the presence of vascular or perineural invasion (Table 1).

### 2.2. Immunohistochemistry

The immunohistochemical techniques were performed on tissue microarrays (TMA), constructed according to a previously described method [16, 19]. For this, two cores (1.5 mm each) from previously selected tumoural areas of each patient were used resulting in three TMA blocks with 134 cores.

For immunohistochemical staining, 4 *μ*m sections from TMA blocks were used. After deparaffinization and rehydration, slides were treated for antigen retrieval and incubated with the primary antibodies listed in Table 2. Visualization was performed using the dextran-polymer system (EnVisionTM Detection Kit, Dako, Glostrup, Demark) and counterstaining with Harris hematoxylin for 2 min. For each staining run, we used positive (tonsil) and negative (omission of primary antibody) controls.

### 2.3. Evaluation of Immunohistochemistry Expression

Two observers (L.M. and M.F.) analysed the immunohistochemically stained slides using an Olympus BX41 microscope, blinded to the clinical characteristics of the tumours. Any discordant case was reviewed under a multihead microscope to achieve a final result. We considered the highest score of the two core disks.

The expression of cyclins was semiquantitatively evaluated on the basis of the extent of nuclear tumour cell staining for cyclin A2, cyclin D1, and cyclin E and cytoplasm tumour cell staining for cyclin B1. This expression was classified on a four-point score: negative (no labelling or labelling in <10% of tumour cells); 1+ (labelling in 10% to 24% of tumour cells); 2+ labelling in 25% to 49% of tumour cells); and 3+ (labelling in 50% or more of tumour cells). In the following high expression, thresholds were used as cut-offs: 25% for cyclin A2, 50% for cyclin D1, and 10% for cyclin B1 and cyclin E1. These cut-offs were determined for each cyclin based on the mean of the expression score for each marker (high value than the mean score was considered high expression) (adapted from Sawair et al. [20]). In this way, a score of 2+/3+ was considered as a high expression for cyclin A2 (mean score of 1.4 ± 0.9), a score of 3+ for cyclin D1 (mean score of 2.3 ± 1.1), and any score of 1+/2+/3+ for cyclin E1 (mean score of 0.8 ± 0.7) and cyclin B1(mean score of 0.4 ± 0.7).

For Ki-67 evaluation, we considered two groups based on nuclear staining of tumour cells: low proliferative tumour (labelling from 0 to 49% of tumour cells) and high proliferative tumour (labelling in 50% or more of tumour cells) [21].

### 2.4. Fluorescence In Situ Hybridization

The detection of 11q13 band was carried out by fluorescence in situ hybridization technique. Briefly, the procedure was as follows:

Slides were dried overnight at 55°C for 20 minutes. After deparaffinization and rehydration, the slides were placed in saline sodium citrate (SSC2x) wash for 3 minutes in a water bath. Paraffin-embedded sample pretreatment was carried out with a sodium thiocyanate (NaSCN) 1 M, at 80°C for 30 minutes. Afterwards, the slides were placed in distilled water for 1 minute and in SSC2x for 5 minutes.

To perform the enzymatic digestion protocol, we used 0.05 mg/mL pepsin solution in HCl 0.01 N. Enzyme activity for the various sections taken from each specimen was stopped at different times by slide immersion in SSC2x for 1 minute and tissue drying in a thermal plate at 45°C. Tissue appearance was then observed under light microscopy with 10x and 40x magnification. Optimal tissue digestion was achieved when the tissue showed fern-like formations under light microscopy. After this, the slides were placed in 2 baths of SSC2x for 3 minutes and dehydrated. Then the slides were air dried at room temperature for 15 minutes.

Probe mix was carried out according to the manufacturer's specifications using the probe CCND1 FISH DNA Probe Split signal (Y5414) (DakoCytomation, Glostrup, Denmark). Hybridization was performed on the DakoCytomation Hybridizer (DakoCytomation Glostrup, Denmark), first at 84°C for 5 minutes and finally at 37°C overnight. For posthybridization washes, we used SSC0.4x with 0.3% Nonidet P-40 (NP-40) at 73°C for 3 minutes. Then the slides were placed in SSC2x with 0.1% NP-40 at room temperature. Afterwards, the slides were air dried for 20 minutes and mounted in DAPI II (4′-6′-diamidino-2-feniloide) counterstain from Vysis Inc. (Downers Grove, IL).

### 2.5. Evaluation of Fluorescence In Situ Hybridization

Image analysis was performed using an Eclipse E400 Nikon fluorescence microscope (Nikon, Tokyo, Japan) equipped with DAPI (nuclei) and Spectrum Green/Spectrum Orange dual band pass filter sets (magnification of 400x). We considered the presence of *CCND1* numerical aberrations when at least 20% of the nuclei exhibited 3 or more signals for *CCND*1 in high-power fields [22].

### 2.6. Statistical Analysis

Possible relations between categorical variables were analysed using chi-square tests. The correlation between protein markers was measured by Spearman's correlation coefficient. Time interval (expressed in months) between primary treatment and last follow-up or death of the patient corresponded to overall survival (OS). Time interval (expressed in months) between primary treatment and the first recurrence (whether local, regional or distant) corresponded to disease-free survival (DFS). Univariate analysis of the influence of markers on five-year OS and DFS was performed using the Kaplan-Meier and the log-rank test. Factors that were significant in the univariate analysis were then analysed by multivariate analysis using the Cox proportional hazards model.

The level of statistical significance was considered at *p* < 0.05.

## 3. Results

### 3.1. Cyclin A2

Of the 134 cores of the cyclin A2 slides, 13 (9.70%) cores could not be analysed: 8 (5.97%) cores contained no tumour cells, 4 (2.99%) were lost, and 1 (0.75%) had not sufficient cells for scoring. In the view of this, 2 (2.99%) out of 67 cases could not be analysed for cyclin A2.

**Figure 1:**
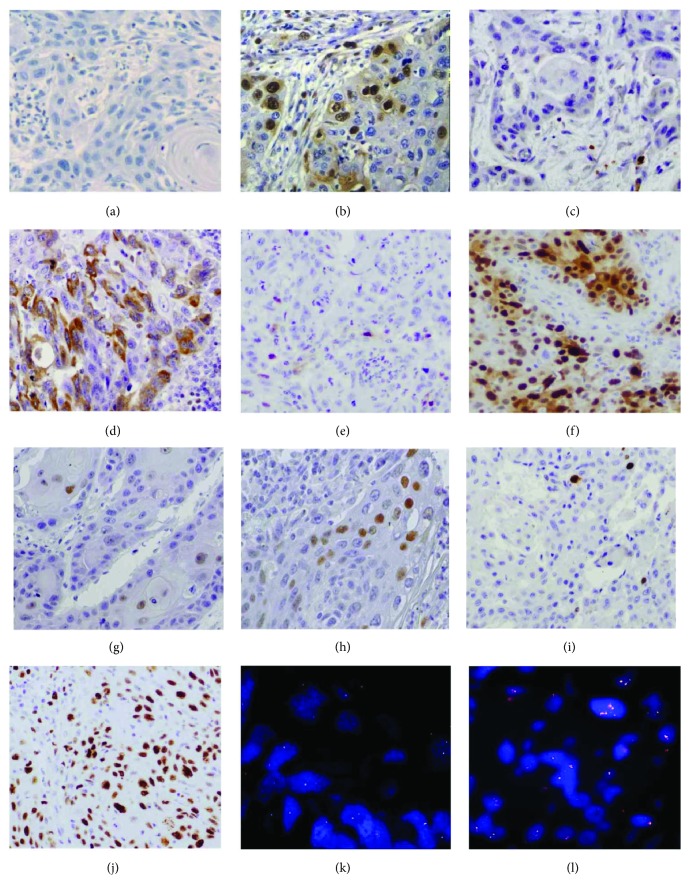
Immunohistochemical staining of cyclins and CCND1 region analysis by fluorescence in situ hybridization technique in OSCC. (a) Low expression for cyclin A2 (magnification ×200). (b) High expression for cyclin A2 (magnification ×200). (c) Low expression for cyclin B1 (magnification ×200). (d) High expression for cyclin B1 (magnification ×200). (e) Low expression for cyclin D1 (magnification ×200). (f) High expression for cyclin D1 (magnification ×200). (g) Low expression for cyclin E1 (magnification ×200). (h) High expression for cyclin E1 (magnification ×200). (i) Low expression for Ki-67 (magnification ×200). (j) High expression for Ki-67 (magnification ×200). (k) Normal *CCND1* numerical signals (magnification ×200). (l) Presence *CCND1* numerical aberrations (magnification ×200).

The cyclin A2 expression was observed in 54 (83.1%) cases (Figures 1(a) and 1(b)), classified as 1+ in 29 (44.6%), 2+ in 15 (23.1%), and 3+ in 10 (15.4%).

### 3.2. Cyclin B1

Out of 134 core disks, 10 (7.46%) cores could not be evaluated for cyclin B1: 8 (5.97%) contained no tumour cells, 1 (0.75%) was lost during processing, and 1 (0.75%) had not sufficient cells for scoring, resulting the elimination of 2 (2.99%) cases.

The cyclin B1 expression was observed in 39 (60%) cases (Figures 1(c) and 1(d)), classified as 1+ in 29 (44.6%) and 2+ in 10 (15.4%).

### 3.3. Cyclin D1

Out of 134 core disks, 10 (7.46%) cores could not be analysed for cyclin D1: 8 (5.97%) contained no tumour cells, 1 (0.75%) was lost during processing, and 1 (0.75%) had not sufficient cells for scoring resulting the elimination of 2 (2.99%) cases.

The cyclin D1 expression was observed in 58 (89.2%) cases (Figures 1(e) and 1(f)), classified as 1+ in 6 (9.2%), 2+ in 13 (20%), and 3+ in 39 (60%).

### 3.4. Cyclin E1

Out of 134 core disks for cyclin E1, 13 (9.7%) cores could not be analysed: 9 (6.72%) contained no tumour cells, 3 (2.24%) were lost during processing, and 1 (0.75%) had not sufficient cells for scoring. Therefore, 3 (4.48%) out of 67 cases could not be analysed for cyclin E1.

The cyclin E1 expression was positive in 21 (32.8%) cases (Figures 1(g) and 1(h)), classified as 1+ in 15(23.4%) and 2+ in 6 (9.4%).

### 3.5. Ki-67

In tissue microarray slides stained for Ki-67, 12 (8.96%) cores could not be evaluated: 7 (5.22%) contained no tumour cells, 4 (2.99%) had too few cells for scoring, and 1 (0.75%) was lost during processing. Because of this, 3 cases (4.47%) could not be analysed at all.

Ki-67 expression was detected in almost all cases (61, 95.3%) except in three. Twenty-nine (45.3%) cases were classified as high proliferative tumours and 35 (54.7%) as low proliferative tumours (Figures 1(i) and 1(j)).

### 3.6. *CCND1* Gene Analysis

Out of 134 core disks, 14 (10.4%) could not be evaluated for *CCND1* analysis: 8 (5.97%) contained no tumour cells and 6 (4.5%) were lost during processing. Therefore, 7 (10.5%) out of 67 cases could not be analysed for *CCND1* gene analysis.

In assessing the *CCND1* gene, we found that 43.3% (*n* = 26) of the cases showed the presence of numerical aberrations (Figures 1(k) and 1(l)). In 19 cases (31.7%), there were more than six signals or cluster formations present *per nuclei*.

### 3.7. Association of High Expression of Markers with Clinicopathological Features

The presence of high expression of cyclin A2 (in 25% or more of tumour cells) was associated with male gender (*p* = 0.023), more advanced T category (*p* = 0.003), and/or more advanced tumour stage (*p* = 0.002). The presence of high expression of Ki-67 was associated with more advanced T category (*p* = 0.003), with more advanced tumour stages (*p* = 0.006), and with the presence of vascular invasion (*p* = 0.016). All cases with vascular invasion were high proliferative tumours.

None of the other markers (cyclin B1, D1, or E1 and *CCND1* gene status) were correlated with clinicopathological features.

### 3.8. Coexpression of Markers of the Study

High Ki-67 expression was observed in tumours with high expression of cyclin A2 (Rho = 0.30; *p* = 0.016) and cyclin B1 (Rho = 0.37; *p* = 0.003). High expression of cyclin A2 was correlated with high expression of cyclin B1 (Rho = 0.32; *p* = 0.009).

Tumours with cyclin D1 high expression (in 50% or more of tumour cells) reached a significant relationship with the existence of numerical aberrations (Rho = 0.48; *p* < 0.001).

### 3.9. Patients' Outcome

**Figure 2:**
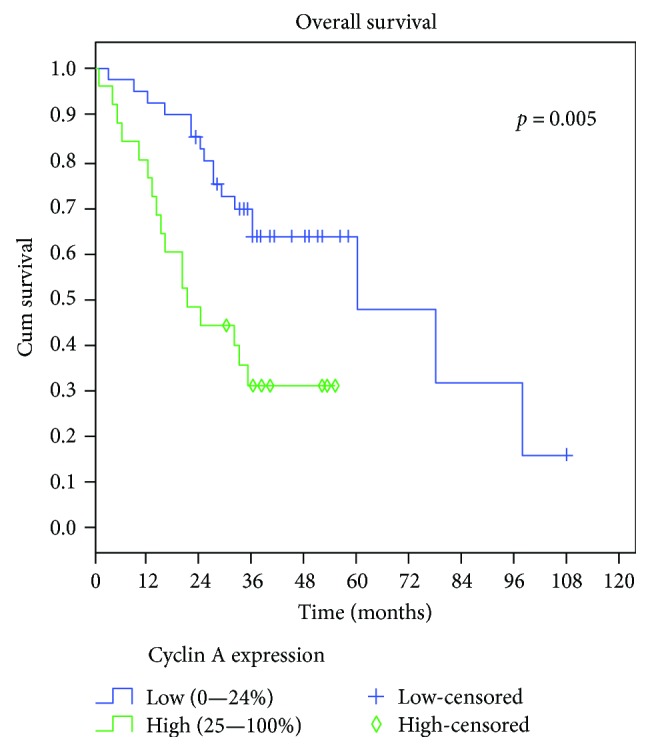
Univariate Kaplan-Meier analysis of overall survival in patients with OSCC. Patients with tumours with high expression of cyclin A2 presented a lower survival rate than patients with low expression of cyclin A2.

Patients were observed for a minimum of 3 years after treatment or until their death. During the follow-up period, 36 (53.7%) had died of oral cancer. Overall survival rate at 5 years of follow-up was 49.4%, and DFS was 34%. In univariate analysis, T status (*p* < 0.001), N status (*p* < 0.001), clinical stage (*p* < 0.001), perineural permeation (*p* = 0.039), and cyclin A2 expression (*p* = 0.005) (Figure 2) were statistically correlated to OS (Table 3). The same was observed with reference to DFS for T status (*p* < 0.001), N status (*p* < 0.001), clinical stage (*p* < 0.001), and tumour grade (*p* = 0.007) (Table 3).

In multivariate analysis for OS, we only found an independent prognostic value for stage and cyclin A2 expression (Table 4), where tumours with advanced stage (*p* < 0.001) and with high expression of cyclin A2 (*p* = 0.031) had lower OS (Table 4). For DFS, advanced stage (*p* < 0.001) and histological differentiation grade (*p* = 0.006) presented adverse independent prognostic value in the multivariate analysis (Table 4).

## 4. Discussion

Deregulation and aberrations of cell cycle-related cyclins have been implicated in the tumour growth and progression of several cancers [3, 5]. The understanding of these alterations in tumorigenesis may identify new proteins that may serve as important cancer diagnostic and prognostic indicators as well as potential targets for therapeutic approaches in patients with OSCC [16, 23]. With this in mind, we conducted this study to evaluate the influence of the expression of the cyclins A2, B1, D1, and E1 and *CCND1* gene status on clinical, pathologic, and prognostic characteristics of patients with OSCC.

Cyclins were highly expressed in the present cohort of OSCC. Cyclin D1 was present in almost 90% of the cases and highly expressed in 60% being the most detected cyclin in these tumours. This is in line with the reported high expression of this protein in OSCC [8, 21, 24]. High expression values were also noted for cyclin A2, observed in more than 80% of tumour cells, and lower frequencies were observed for cyclins B1 and E1. These levels of expression are in accordance with the reported literature for these proteins reflecting the relevant role of these proteins in OSCC [6, 11, 13, 25].

Several clinical and pathological indicators of the tumour progression, such as tumour size, nodal metastasis, clinical stage, and histological grade, have been related with cyclin expression. This is in accordance with the crucial contribution of cyclins in control of the cell cycle and thereby tumour proliferation [6, 11, 22]. We could confirm the associations of cyclin A2 with tumour size (T), nodal metastasis, and the clinical stage suggesting that high expression of cyclin A2 could be relevant for tumour progression and metastization. Chen et al. [11] observed a significant association between high cyclin A2 expression and advanced stage tumour, advanced tumour size (T), and presence of nodal metastasis. Saarilahti et al. [6] reported an association with histological grade.

To assess the relation of the expression of these cyclins with proliferative tumour phenotype, we evaluated the expression of the nuclear protein Ki-67, a proliferation marker described in some studies as an indicator of aggressive behaviour serving as a prognostic indicator [21]. In the present work, high Ki-67 expression was detected in almost half of the cases and related with T and clinical stage and also with the presence of tumour vascular invasion. Interestingly, Ki-67 was significantly associated with the expression of cyclin A2 and cyclin B1 confirming the proliferative capacity of these tumour cells. Other authors have also observed this association between cyclin A2 and Ki-67 [6, 13, 26].

Cyclin D1 overexpression has been related with amplification of the CCND1 gene in several tumours including OSCC [22, 27, 28]. The activation of the pathways involved in cyclin D1 expression appears to be essential in the development of human cancers, including oral cancer [27]. In view of this, we analysed the status of the 11q13 region where this gene is located. We observed that 43.3% of the cases presented aberration of this region with 6 or more signals per cell in 32% cases. Similar values were reported by other authors [7, 22, 29] in head and neck cancer.

Furthermore, we observed a significant association of CCND1 status with cyclin D1 high expression. These results suggest that overexpression of cyclin D1 could be caused by numerical aberrations in the 11q13 region such as CCND1 amplification. Nevertheless, there were some cases with cyclin D1 high expression that presented a normal 11q13 status. This suggests that mechanisms other than amplification of CCND1 gene could be involved in overexpression of this cyclin including increasing mRNAs, decrease in cyclin D proteolytic degradation or DNA methylation [30, 31].

An important aspect of a molecular marker is the relation of the marker with the prognosis of the patient. We observed that OSCC patients with high expression of cyclin A2 in their cell tumours presented a significantly lower 5-year overall survival even in multivariate survival analyses, indicating that cyclin A2 expression could be an independent prognostic marker in OSCC. Chen et al. [6], evaluating squamous cell carcinomas of the oral cavity, found an association between high cyclin A2 expression and lower overall survival. This was also seen in the study of Saarilahti et al. [11], in addition to the association with disease-free survival although in laryngeal carcinomas. Thomson et al. [32] observed an association of cyclin A expression with the recurrence of the disease in malignant and premalignant lesions of the oral cavity. Cyclin A is required for DNA synthesis during the S phase and progression through the G2/M transition. Our results suggest that tumours with cyclin A2 expression will drive the cells to division thus rendering them some growth potential. This will influence the survival/death of the tumour in patients with overexpression of this protein marker. In our series, no other cyclins or markers studied were significantly related with survival. Nevertheless, some studies reported the influence of other cyclins such as cyclin D1 [8, 10, 22, 29, 30, 33] or cyclin B1 [25] on the prognosis of OSCC. Some recent papers have suggested the influence of CCND1 amplification on a poor overall survival and cervical nodal metastization which led us to evaluate not only the protein expression of cyclin A2 but also the CCND1 region status [7, 9, 22]. We could not find an association of numerical aberrations in the 11q13 region such as CCND1 amplification with any clinical and pathological variables such as nodal metastasis and also with survival. This could be due to the small size of our series, differences in tumour sites [7], or possible differences in geographic populations [8, 10]. Our data indicate that for South European populations, cyclin A2 is a major prognostic marker, being an indicator of high proliferative status tumours but importantly also of a poor patient's survival with these tumours. This can assist oncologists in the planning treatment options for each patient with OSCC, indicating better and more adjusted chemotherapy or radiotherapy protocols considering the cyclin A2 status of the tumours [34, 35]. Moreover, these proteins could function as targets for molecular therapies against this pathway leading to chronic mitotic arrest and cell death by apoptosis [36]. Our findings thus could have a translational value in the development of targeted therapies for OSCC [37].

Nevertheless, we wish to highlight some limitations of the present study, related with its retrospective nature that sometimes could not give the range of variables and information that prospective studies could give.

In conclusion, we report high expression of cell cycle-related proteins such as cyclin D1 and cyclin A2 in OSCC. Importantly, we found an independent prognostic value for cyclin A2 protein in OSCC indicating a poor overall survival for patients with tumours with high expression of cyclin A2. The present study suggests that this protein could serve as a prognostic marker in OSCC.

## Figures and Tables

**Table 1:** Patient characteristics (*N* = 67).

Factor	Group	*N* (%)
Gender	Female	15 (22.4%)
	Male	52 (77.6%)

Age (mean age: 59 ± 12.6 years)	<59 years	33 (49.3%)
	≥59 years	34 (50.7%)

Location	Tongue	28 (41.8%)
	Floor of the mouth	20 (29.9%)
	Gingiva	7 (10.4%)
	Retromolar trigone	4 (6.0%)
	Hard palate	5 (7.5%)
	Buccal mucosa	3 (4.5%)

Tumour size	T1	22 (32.8%)
	T2	27 (40.3%)
	T3	6 (9.0%)
	T4	12 (17.9%)

N status	N0	46 (68.7%)
	N1	11 (16.4%)
	N2	10 (14.9%)

Stage	I	21 (31.3%)
	II	20 (29.9%)
	III	11 (16.4%)
	IV	15 (22.4%)

Treatment^∗^	SG	49 (73.1%)
	SG + RT	18 (26.9%)

Tumour grade	G1	34 (50.7%)
	G2	29 (43.3%)
	G3	4 (6%)

Margin status^∗∗^	Free of tumour	54 (85.7%)
	With tumour	9 (14.3%)

Vascular invasion	Absent	62 (92.5%)
	Present	5 (7.5%)

Perineural permeation	Absent	56 (83.6%)
	Present	11 (16.4%)

SG: surgery; RT: radiotherapy (consisting in adjunctive external-beam radiotherapy, 55-66Gy). ^∗^Patients were excluded if they had undergone radiotherapy or chemotherapy prior to surgery. None of the included patients received molecular therapies against EGFR or other proteins. ^∗∗^Not available in 4 cases. Table 1 is reproduced from Monteiro et al. [16] (under the Creative Commons Attribution License/Public Domain).

**Table 2:** Primary antibodies used in the present study.

Antibody	Clone	Dilution	Pretreatment	Manufacturer
Anti-Ki-67	MIB-1	1/200	WB + TE	DakoCytomation, Glostrup, Denmark
Anti-cyclin D1	SP4	Prediluted	WB	Master Diagnostica, Spain
Anti-cyclin E1	13A3	1/50	WB	Novocastra Leica Biosystems, Newcastle upon Tyne, UK
Anti-cyclin A2	6E6	1/10	WB	Novocastra Leica Biosystems, Newcastle upon Tyne, UK
Anti-cyclin B1	7A9	1/10	WB	Novocastra Leica Biosystems, Newcastle upon Tyne, UK

WB: waterbath at 98°C 30 minutes; TE: tris-ethylenediaminetetraacetic acid (EDTA).

**Table 3:** Univariate analysis of overall survival and disease-free survival at 5 years, according to clinicopathological characteristics and marker expression.

Factor	Group	*N*	Overall survival (5 years)	*p* value	*N* ^∗∗^	Disease-free survival (5 years)	*p* value
Gender	Female	15	64.2	0.206	14	50	0.442
	Male	52	47.3		50	30.7	

Age	<59 years	33	44.4	0.500	33	30.6	0.534
	≥59 years	34	54.6		31	39.6	

Location	Tongue	28	46.5	0.261	28	44.5	0.688
	Floor of the mouth	20	44.4		20	25	
	Gingiva	7	28.6		5	40	
	Retromolar trigone	4	50		3	33.3	
	Hard palate	5	80		5	20	
	Buccal mucosa	3	0		3	66.7	

Tumour size	T1	22	71.5	<0.001	22	58.3	<0.001
	T2	27	53.2		27	30.5	
	T3	6	33.3		6	22.2	
	T4	12	8.3		9	0	

N status	N0	46	68.2	<0.001	46	44.9	<0.001
	N1	11	9.1		10	12	
	N2	10	10		8	0	

Stage	I	21	74.9	<0.001	21	61.1	<0.001
	II	20	67.8		20	43.3	
	III	11	27.3		11	21.2	
	IV	15	6.7		12	0.0	

Treatment	SG	49	53.2	0.340	48	34	0.964
	SG + RT	18	38.9		16	35.7	

Tumour histological grade	G1	34	63.7	0.147	32	47.3	0.011
	G2	29	36.1		28	27.2	
	G3	4	25		4	0	

Margin status^∗^	Free of tumour	54	51.9	0.211	53	37.8	0.757
	With tumour	9	33.3		8	37.5	

Vascular invasion	Absent	62	50.1	0.620	59	33.9	0.886
	Present	5	40		5	40	

Perineural permeation	Absent	56	54	0.039	54	34.2	0.632
	Present	11	27.3		10	37.5	

Cyclin A2	<25%	40	63.3	0.005	40	43.8	0.203
	≥25%	25	30.8		22	22.3	

Cyclin B1	<10%	26	57	0.331	25	32	0.526
	≥10%	39	46.9		37	39.2	

Cyclin D1	<50%	26	60.6	0.172	24	49.4	0.236
	≥50%	39	44.8		38	26.3	

Cyclin E1	<10%	43	48.2	0.233	40	33.9	0.758
	≥10%	21	59.1		21	41.6	

Ki-67	<50%	35	58.6	0.111	35	38.1	0.988
	≥50%	29	44.0		26	36.1	

CCND1	Normal	34	52.3	0.991	32	40.2	0.412
	Numerical aberration	26	47.1		25	29.7	

SG: surgery; RT: radiotherapy. ^∗^Not available in 4 cases. ^∗∗^The number of patients in the disease-free survival in some of the analyses is different from the overall survival analysis as some patients had persistence of the disease and were only evaluated in the overall survival.

**Table 4:** Variables included in the multivariate Cox regression analysis of overall survival and disease-free survival.

Factor	Group	N	*p* value	Exp (*β*)	95% CI of Exp (*β*)
		*Overall survival*			
Tumour size	T1	21	0.659	1	
	T2	27	0.263	0.223	0.016–3.08
	T3	6	0.368	0.237	0.01–5.445
	T4	11	0.217	0.220	0.2–2.437

Nodal status	N0	45	0.716	1	
	N1	11	0.466	1.672	0.42–6.658
	N2	9	0.462	1.909	0.341–10.678

Stage	I	20	<0.001	1	
	II	20	0.922	1.063	0.315–3.58
	III	11	0.041	3.548	1.052–11.963
	IV	14	<0.001	14.309	4.641–44.121

Perineural permeation	Absent	54	0.306	1	
	Present	11		1.654	0.631–4.339

Cyclin A2	<25%	40	0.031	1	
	≥25%	25		2.397	1.085–5.295

		*Disease-free survival*			
Tumour size	T1	22	0.407	1	
	T2	27	0.783	0.691	0.05–9.568
	T3	6	0.426	0.281	0.012–6.396
	T4	9	0.234	0.249	0.025–2.455

Nodal status	N0	46	0.484	1	
	N1	10	0.367	0.479	1.1–5.362
	N2	8	0.232	0.313	1.821–19.42

Stage	I	21	<0.001	1	
	II	20	0.232	1.741	0.7018–4.321
	III	11	0.054	2.666	0.982–7.237
	IV	12	<0.001	9.767	3.727–25.599

Histological grade	G1	32	0.006	1	
	G2	28	0.028	2.428	1.100–5.362
	G3	4	0.003	5.947	1.821–19.420

Variables included in multivariable Cox regression analysis for OS: tumour size, nodal status, clinical stage, perineural permeation, and cyclin A2 expression, and for DFS: tumour size, nodal status, clinical stage, and tumour histological grade.
